# A chronological discourse analysis of ancillary care provision in guidance documents for research conduct in the global south

**DOI:** 10.1186/s12910-022-00789-6

**Published:** 2022-05-14

**Authors:** Blessings M. Kapumba, Nicola Desmond, Janet Seeley

**Affiliations:** 1 London School of Hygiene and Tropical Medicine, London, UK; 2 Malawi-Liverpool Wellcome Trust Clinical Research Programme, P.O. Box 30096, Chichiri, Blantyre 3, Malawi; 3 Liverpool School of Tropical Medicine, Liverpool, UK

**Keywords:** Ancillary care, Discourse analysis, Ethics guidelines, Policy documents, Health-related research, Resource-constrained settings, Malawi

## Abstract

**Introduction:**

Numerous guidelines and policies for ethical research practice have evolved over time, how this translates to global health practice in resource-constrained settings is unclear. The purpose of this paper is to describe how the concept of ancillary care has evolved over time and how it is included in the ethics guidelines and policy documents that guide the conduct of research in the global south with both an international focus and providing a specific example of Malawi, where the first author lives and works, as a case study.

**Methods:**

Discourse analysis was conducted on 34 international ethics guidelines and policy documents. Documents were purposively selected if they contained a set of key terms that reflect the concept of ancillary care. Following a process of inductive discourse analysis, five key interrelated text phrases relating to ancillary care were extracted from the documents. The evolution of these phrases over time was explored as they represented the development of the concept of ancillary care as a component of ethical health research guidance and practice.

**Results:**

We found key interrelated phrases that represent discourses regarding the evolution of ancillary care including participant protection; provide care as appropriate; supererogation; patient needs prevail over science; and ancillary care as an obligation. Arguments for the provision of ancillary care were characterised by safeguarding the safety, health rights and well-being of study participants. However, despite the evolution of discourse around ethical obligations to provide ancillary care, this is rarely made explicit within guidance documents, leaving interpretive space for differential application in practice.

**Conclusion:**

While there have been major changes to the ethics guidance that reflect significant evolution in the ethical conduct of research, the specific vocabulary or language used to explain the ethics of researchers' ancillary care obligations to the health needs of their research participants, lacks clarity and consistency. As a result, the concept of ancillary care continues to be under-represented in local ethical guidelines and regulations, with no clear directives for country-level research ethics committees to apply in regulating ancillary care responsibilities.

## Introduction

Numerous guidelines and policies for ethical research practice have evolved over time. The abuse of study participants in early experiments, a violation of human rights as spelt out in article 25 of the UN General Assembly [1], triggered the development of guidelines and regulatory policies for human research ethics. Many guidelines and regulatory policies have been developed in response to historical abuses of human participants in experiments, such as the Nazi research on prisoners which led to changes in research guidance and practice [2, 3]. The learning from lengthy international consultative processes which have taken place over recent decades resulted in the development of further guidance by the World Medical Association [4], the Belmont Report by the National Commission for the Protection of Human Subjects of Biomedical Behavioral Research [5], the International Conference on Harmonisation Guideline for Good Clinical Practice (ICH GCP) [6] (not per se an ethics guideline but an international ethical and scientific quality standard commonly used as the basis for ethics and ethical decision making in health-related research that involve the participation of human subjects), and the Council for International Organisation of Medical Sciences (CIOMS) [7]. In developing these research ethics guidelines, the primary focus was to ensure the safety and well-being of research participants to prevent the reoccurrence of historical abuse. Relatively limited attention has been paid to the genesis of these texts and how certain aspects of the guidance have evolved over time and how this evolution in language has influenced the emphasis on different aspects of ethical conduct of research in different contexts.

International research ethics guidelines and policies now espouse the commitment of researchers to serving the participants who volunteer for research by being responsive to their health needs, both as a direct result of research participation and more broadly as an ethical obligation [8, 9]. Increasingly, these ethical guidelines emphasise optimal health benefits for research participants. The extent to which these guidelines are adhered to, especially when research is undertaken in resource-constrained settings, has increasingly formed a significant component of this discourse. According to these discussions, while the provision of care to study participants appears to be broadly recognised in international ethics guidelines such as the CIOMS [7], the World Medical Association Declaration of Helsinki [9], and the ICH GCP [10], its implementation has been slow, making the universality of these guidelines problematic.

Driven by a global discourse prioritising the rights of research participants in the ethics of health research practice, the concept of ancillary care has become increasingly common in medical research. Recent discussions (triggered by Belsky and Richardson [11]) highlight the body of literature available on the provision of care during medical research, but does not focus on how the central ethical concept of providing for the ancillary health needs of research participants became increasingly important [12]. Participants and communities in low resource settings where global health research takes place increasingly demand protection and care from researchers. The provision of ancillary care in low-resource settings may be advocated under a human rights approach that supports and strengthens medical research ethical standards of conduct and adds to the global scientific debate on ethics [13, 14]. Particularly, ancillary care concerns broaden appreciation of the critical nature of protecting the rights of study participants and the extent to which researchers demonstrate an ethical commitment to their subjects.

Richardson [12], Hyder, Merritt [15], Merritt [16], and Pratt et al. [17] have critically examined the basis for the need for ancillary care to be provided to study participants by researchers in medical research. The authors have emphasised three tenets for the provision of ancillary care related to: researchers special duty to care [18], partial-entrustment [11] and principles of justice [19]. Whilst these arguments are coherent as providing principles for ancillary care provision, there remains scant guidance on how this should practically be provided when medical research is undertaken in resource-constrained settings with no or limited availability of care in the communities where participants live, and typically without viable and functioning services for alternative treatment options.

Our earlier research on current practices of ancillary care in East and Southern Africa demonstrated that care and support for study participants during medical research remain lacking, with no standardized guidelines [20]. Furthermore, there are contextual factors in resource-constrained communities in the global south that impact the decision regarding participation in health-related research, such as gaining access to better health care services. Given this, most research ethics committees (REC) in these settings lack the proper guidance to assess the issue of ancillary care in context. Specific guidelines should be available for those who are tasked with making these decisions.

Bringing together evidence of ancillary care from international and local ethics guidelines and policies, the analysis presented in this paper provides a part of an evolving process that aims to develop specific ethics guidelines for ancillary care in medical research and its application in resource limited settings. The purpose of this paper is to describe how the concept of ancillary care has evolved over time and how it is included in the ethics guidelines and policy documents that guide the conduct of research in the global south with both an international focus and providing a specific example of Malawi, where the first author lives and works, as a case study. This paper builds on the work done by Krubiner et al. [21] but focuses explicitly on how the language surrounding the provision of ancillary care has changed over time. We trace the documents backwards to look at where the influences on ancillary care were and how that has influenced or impacted on the ethics of medical research in practice. Specifically, we describe what is defined in the research ethics guidelines and policies regarding researchers’ responsibilities towards their participants, we document the chronology of how the concept of providing care to study participants has evolved over-time and through this, explore how the ethics of ancillary care has been justified within guidance and policy documents for practice.

## Methods

### Design

To develop an understanding of how the concept of providing care to study participants has evolved over time, we used discourse analysis to interrogate a purposively selected sample of research ethics guidelines and institutional policy documents. We examined how unique discursive features of guideline documents contribute to the construction of ancillary care in medical research. Critical discourse analysis is a technique for exploring the links between discursive texts, events, and practices, as well as wider social and cultural structures, relationships, and processes [22]. In this study, it was used to determine how ancillary care is shaped by different research ethics guidelines and policies over time. According to Van Dijk [23] discourse analysis seeks to reveal implicit and hidden power dynamics enacted in discourse, as well as the various discursive strategies of dominance and resistance. Due to the lack of clarity on these relationships, it is probable that those responsible for developing these guideline documents may be unaware of the connections between ancillary care provision, power dynamics in research, and discourse.

### Document selection for analysis

This study involved the collection and analysis of research ethics guidance and policy documents relevant for the ethical practice of medical research globally. The use of ethics guidelines and policy documents as a framework to evaluate the idea of ancillary care was considered because they directly dictate the ethical conduct of medical research involving human subjects. Additionally, these documents were chosen for this study because they provide ethical framework for scientifically and ethically sound medical research.

We conducted a search for and purposively selected the main international ethics guidance documents that are used as guidance for the conduct of medical research, including the Nuremberg code, the Declaration of Helsinki, the Belmont report, the ICH-GCP, and the CIOMS. We traced the emergence of guidance within the international ethics guidelines across time, based on the chronology of their publication made available on their official websites, for example, the World Medical Association, ICH-GCP, and CIOMS websites. Additional documents were included if they were mentioned or cited in already-included documents or secondary literature on the subject, and that they provide ethical guidance on the conduct of health research in resource-constrained settings (RCS) such as the Nuffield Council on Bioethics report. For the funding agencies, ethical guidelines and policy documents were obtained directly from organization or institution websites or, if the organisation did not make them available, from the regulatory authority that published the document. We sought to get access to documents from funding agencies that we considered could have such guidelines and policies because they fund large-scale research projects in resource-constrained settings including in Malawi (Table 1). From the local regulatory institutions, BK requested for a collection of guidance documents by asking directly from local institutions to suggest ethics or policy documents that are used as guidelines, and which are not available online. When requesting for the documents, members of the institution or organisation were asked to suggest ethics or governance documents that could provide principles to guide the conduct of medical research. In total, we reviewed 88 ethics documents to determine their length, genre and primary objective or focus.

**Table 1:** Inclusion criteria for funding organisations, research institutions and documents

Selection of research funding organisation	Selection of local (Malawi) research institutions and regulatory bodies	Inclusion criteria of document
Those directly provide funding for research studies in RCS (Malawi)	Involved in reviewing and approving study plans, and monitor study progress—Research Ethics Committees	The ethics guidelines and policy or regulatory documents were included if they contained statements with key phrases that represent various discourses that could imply the provision of ancillary care to research participants
	Conducts a wide range of research projects including clinical trials—Malawi-Liverpool Wellcome Trust Clinical Research programme	Protection of participants rights, safety, life, health, and well-being
		Preparations or plans for participant’s care
		Respect for participants rights and integrity
	Responsible for regulation of all health research conducted in Malawi—Malawi Ministry of Health Research department	Care as an act of kindness
		Beneficence
		Participant interests considered first
		Responsiveness of research towards participants health needs
	Responsible for the development of research ethics guidelines—National Commission for Science and Technology	Researchers’ responsibility or duty to care
		Morally praiseworthy
		Provide care as appropriate, feasible, or necessary
		Participants care obligation

**Fig. 1:**
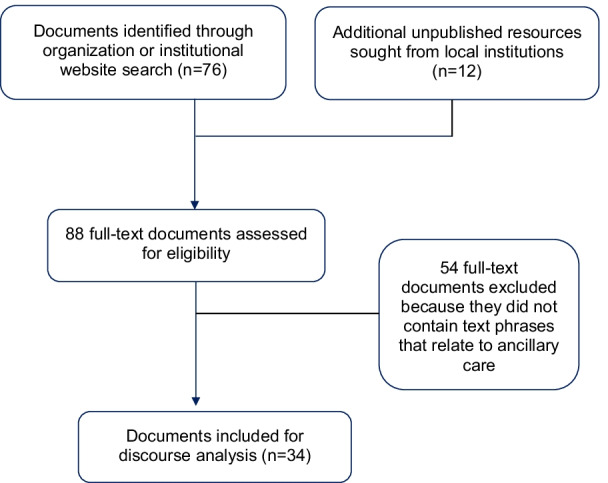
Document identification, screening, and selection

In the second phase, we applied discourse analysis to 34 documents that had key textual phrases related to ancillary care, these were then included in the final analysis (see Fig. 1). From the international ethics guidelines on the conduct of research involving human subjects we included 18 documents; 10 other documents included were for international financing organisations; and, in order to focus on our country case study, 6 guidelines and policy statements were from the Malawi research institutions and regulatory bodies such as the Malawi National Health Sciences Research Committee (NHSRC).

Selected supportive and supplemental resources were included if they related to provision of care or support to study participants. The selection was restricted to documents in English language. Documents that discussed general ethical principles of medical research, without explicit mention of the concepts related to provision of care to study participants, were excluded.

The first document we chose to review was the Nuremberg Code [2], an important guidance document with a global/universal ethics focus. A fundamental principle of the Nuremberg Code was the recognition of the dignity of the individual, which was also the cornerstone of the Universal Declaration on Human Rights [1]. Second, we included documents that were developed following the Nuremberg Code, including the Declaration of Helsinki [4, 9, 24, 25], the Belmont Report [5], CIOMS [7, 8, 26, 27], the ICH-GCP [6], and the Nuffield Council on Bioethics [28]. We traced these international research ethics guidance documents chronologically. The final selection of included documents was for those from funding agencies including Wellcome Trust, European & Developing Countries Clinical Trials Partnership (EDCTP), National Institute of Health (NIH), National Institute for Health Research (NIHR), Medical Research Council (MRC). We also included local (Malawi) research ethics guideline and policies including the research ethics committee guidelines, research institutions (Malawi-Liverpool Wellcome Trust) policies, ministry of health research policies, mainly to look at what documents they refer to and to see their wording for provision of care to study participants.

### Analysis

The selected documents were coded iteratively in NVivo (QSR, Melbourne) by BK. During the first coding process, the texts were reviewed several times starting with the Nuremberg code, paying attention to words, phrases, and concepts related to the provision of care and support to study participants during medical research and exploring how these changed over time. During the second step of the analysis of subsequent documents, we used the key phrases that had been identified for coding while also identifying new phrases that related to ancillary care. We also looked at the general structure, which included the formatting and their order, the use of quotes to introduce specific aspects, and the overall tone and verb tense of the text. We were particularly interested in tracing the use of such phrases in various research ethics guidelines and policies, as well as how the language has evolved over time. In the final stage of the analysis, the coded text phrases were read and key themes (described in the findings section) that best describes the discourses around the provision of ancillary care to study participants and the related ethical justification were generated.

The analysis followed a framing used by Johnstone [29] for discourse analysis, which takes multiple facets of a text into account simultaneously. Six factors are included in this framing: the medium (print or video), the language (particular word choices), the people or participants represented, the author's objectives or purpose, and the social and cultural context. As previously noted, representations of care provision are critical textual targets for this study, and as a result, we focused on ancillary care or guidance for the provision of care to study participants during medical research. We asked how documents explicitly or implicitly explain ancillary care and how does it appear to be referred to (for example, what ethical and other justifications apply?).

All authors discussed the interpretations during regular meetings and there was congruence among the findings that emerged. Therefore, the interpretation provided in this article is based on a critical discourse analysis of texts relevant to the ethics of ancillary care as described in international and local research ethics guidelines and policies.

### Findings

Different constructions of the research ethics guidance documents were reflective of the discourses around the idea of ancillary care. The documents differed profoundly in how they characterised the evolution of provision of care to study participants. We illustrate these findings using relevant quotes describing each of the analysed texts separately and sequence from broad international ethics guidelines (the Declaration of Helsinki, Belmont Report, ICH-GCP, and CIOMS) to specific local research ethics guidance documents [30–33], and international funding agencies policies. We use key interrelated phrases extracted from guidance documents to illustrate the findings: participant protection; provide care as appropriate; supererogation; patient needs prevail over science; and ancillary care obligation. These phrases reflect defined views on ancillary care that have been included into ethical guidelines and policy statements for use in health-related research globally. We discuss how these extracts have been put into the context of research ethics over time and how they relate to ancillary care (Table 2).

**Table 2:** summary of specific phrases reflecting broader discourse

Document Source and year of publication	Title	Discourses				
		Participant protection	Supererogation	Participant needs prevail over science	Provide care as appropriate	Ancillary care researcher’s obligation
Nuremberg Code [2], 1947	Permissible medical experiments	Protect participants against study related harm				
World Medical Association [4], 1964	Human Experimentation: Code of Ethics of the World Medical Association (Declaration of Helsinki)	Researchers protect life and health of the participant		Respect the right of participants to safeguard their integrity		
National Commission for the Protection of Human Subjects of Biomedical Behavioral Research [5], 1978	The Belmont report: ethical principles and guidelines for the protection of human subjects of research	Secure participants well-being				Beneficence (act of kindness) as an obligation
Council for International Organizations of Medical Sciences [51], 1991	International ethical guidelines for review of epidemiological studies	Protect the rights and assure the welfare of subjects			Where participants need health care, arrangements should be made to have them treated or they should be referred to a local health service	
International Conference on Harmonisation—Guideline for Good Clinical Practice [6], 1996	International Conference on Harmonisation of technical requirements for registration of pharmaceuticals for human use	Protect rights, safety, and well-being of participants		Participants rights, safety, and well-being prevail over science	Provide adequate care for study related conditions	Provide care be considered for intercurrent conditions
World Medical Association [24], 2000	Ethical principles for medical research involving human subjects (Declaration of Helsinki)	Protect the life, health, privacy, rights, and dignity of participants		Participant well-being to take precedence over science	Providing care as combined with research	Responsibility for the human subject must always rest with a medically qualified person and never rest on the participant
World Health Organization [42], 2000	Operational guidelines for ethics committees that review biomedical research	Safeguarding the dignity, rights, safety, and well-being of participants		Research interests should not override the health, well-being, and care of research participants	Provide care to research participants during and after the course of the research	
Coucil for International Organisation of Medical Sciences [7], 2002	International ethical guidelines for biomedical research involving human subjects	Protect the rights and welfare of vulnerable persons	Morally praiseworthy for researchers to provide ancillary care to participants		For ancillary health needs researchers should, as appropriate, advise them to obtain, or refer them for, medical care	
Nuffield Council on Bioethics [28], 2002	The ethics of research related to healthcare in developing countries	Protect participants from hard in RCS			Where it is feasible researchers have a duty to provide care for ancillary health needs	
Medical Research Council [41], 2004	MRC Ethics guide: Medical research involving children			Participants' interests must prevail over those of science		
World Medical Association [38], 2004	Declaration of Helsinki: Ethical Principles for Medical Research Involving Human Subjects	Protect participants health, life, privacy, and dignity		The well-being of the participants must take precedence over all other interests		
Medical Research Council [40], 2007	MRC Ethics guide: Medical research involving adults who cannot consent			Respect the interests of an individual participant is more important than any potential benefits of the research to others		
Malawi National Health Sciences Research Committee [30], 2007	General Guidelines on Health Research				Provide care to research participants during and after the course of the research	
World Medical Association [25], 2008	Ethical principles for medical research involving human subjects (Declaration of Helsinki)	Protect the life, health, dignity, integrity, right to self-determination, privacy, and confidentiality of personal information of research subjects		The well-being of the individual research subject must take precedence over all other interests		
Council for International Organisations of Medical Sciences [26], 2009	International ethical guidelines for review of epidemiological studies		Morally praiseworthy for researchers to provide ancillary care to participants			
College of Medicine Research Ethics Committee [31], 2010	General guidelines on health research	Promote dignity, rights, safety, and well-being of research participants				
Malawi Ministry of Health [32], 2012	National Health Research Agenda 2012–2016	Protect and promote the dignity and rights of all research participants				
World Medical Association [9], 2013	Ethical principles for medical research involving human subjects (Declaration of Helsinki)	Promote and safeguard the health, well-being, and rights of participants		The goal of research should never take precedence over the rights and interests of individual research subjects		
Council for International Organisations of Medical Sciences [8], 2016	International ethical guidelines for health-related research involving humans				Make adequate provisions for addressing participants’ health needs during research and, if necessary	
Health Research Authority [43], 2017	UK policy framework for health and social care research	Ensuring participants’ safety and well-being in relation to their participation in the research		Safety and well-being of the individual prevail over the interests of science		
ICH E6(R1) Good Clinical Practice ICH E6(R2) ICH Consensus Guideline [10], 2016	Integrated addendum to ICH E6 (R1): guideline for good clinical practice E6 (R2)	Protect rights, safety, and well-being of participants				When the investigator becomes aware of an intercurrent condition, should notify the participant
Wellcome Trust [48], 2018	Good research practice guidelines	protect the rights, interests and safety of research participants				
H3Africa [47], 2018	Guideline for the Return of Individual Genetic Research Findings				Depending on clinical validity and relevance, advisable to provide referral as ancillary care	
Ministry of Health and Population [33], 2019	National Health Research Policy: Strengthening health research to improve national health security	Protect the rights of research participants				
Wellcome Trust [44], 2020	Research involving human participants policy	Protect the rights, interests and safety of participants			Provision of care as collateral benefits of carrying out research, whether or not they are necessary for the research design	
Council for International Organisations of Medical Sciences [27], 2021	Clinical research in resource-limited settings. A consensus by a CIOMS Working Group					Researchers have an ethical obligation to care for participants’ health needs during research, if necessary
Guenter et al. [52], 2021	Ethical considerations in HIV prevention trials: Joint United Nations Programme on HIV/AIDS and the World Health Organization	Researchers to take measures to protect the safety, dignity, human rights and welfare of participants				
National Institutes of Health [49], 2021	National Institute of Health Grants Policy Statement	Protect the rights and welfare of these participants				NIH-funding for research projects may include for costs towards participants hospitalisation, testing, or care services

### Participant protection

The one thing that all the different guidelines and policies have in common is that of safeguarding the safety of study participants from undue risks of harm. The discussion around the protection of study participants is based on the established ethical principles that grew out of the ethical condemnation of Nazi experiments [2] and the philosophical underpinnings of ethical debates on justice and moral obligation [34, 35]. In the context of this paper, we found that, across all ethical guidance documents derived from the Nuremberg code, the protective obligation of researchers towards their participants is confined to study-related harm. Using the word "protectionism," Moreno [36] explains the ethical need to protect that is outlined in the ethics guidelines. In Moreno's description, protectionism is a concept that emphasises the need of protecting human subjects from the risks associated with involvement in research. This is founded on the concept that a special duty is owned to those who participate in research. This is the case for both international guidelines and their interpretation within funding requirements, regulatory bodies, and research ethics committees which refer exclusively to protection of human research participants and place a strong emphasis on study-related harm.

In 1947, the Nuremberg code [2] established the first international guideline, stating that any study involving human participants must guarantee that adequate safeguards against experiment-related harm are made available. The Nuremberg code's participant protection provisions were wide, including even improbable risks of injury, impairment, or death. The Nuremberg code was the first guideline to put a high value on safeguarding people from research-related harm. Following that, research ethics guidelines were developed to strengthen that protection, through the Declaration of Helsinki, for example, which emphasises the protection of the well-being of research participants as being more important than the research results. In 1964, the World Medical Association Declaration of Helsinki [4] widened the scope of the protective duty to include specifically text on the life and health of participants.

It is the duty of the doctor to remain the protector of the life and health of that person on whom clinical research is being carried out [4].

By adding a broader term such as "protection of the participant's life and health," attention may have been given to caring for any conditions that the participant may be suffering from while participating in the study, thus broadening the scope of responsibility assigned to those seeking to recruit participants in research. However, the focus remained on study-related issues, with little or no mention of care for additional health needs. Later, in the Belmont Report of 1978 [5], another broad idea of protection for study participants was emphasized, in which protection would be targeted at the overall well-being of a person who is involved in research. The Belmont Report went on to establish an additional idea of well-being, which corresponded to what is included in the World Health Organization's 1948 definition of health, “a state of complete physical, mental and social well-being and not merely the absence of disease or infirmity” [37].

Persons are treated in an ethical manner not only by respecting their decisions and protecting them from harm, but also by making efforts to secure their well-being [5].

**Fig. 2:**
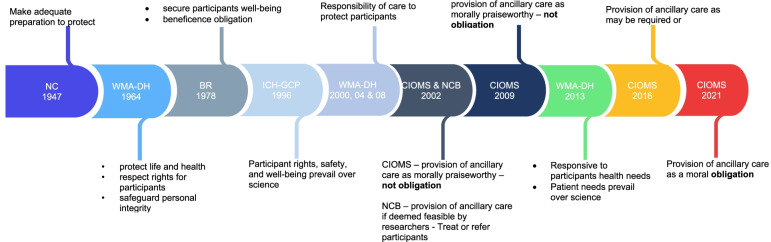
key phrases that reflect the evolution of providing care to participants

The above description of the range of protective duty of researchers towards their participants has evolved over time and used differently in guidance documents, however, the concept remains to refer to ensuring the safety of study participants. From the Nuremberg code which was concerned with the protection of experimental subjects (participants) from study related harm, the concept has evolved through different international ethics guidelines. Several other ethics guidelines have focused on the protection of the life and well-being of study participants for example, the Belmont Report, and the CIOMs (Fig. 2).

The recently updated guidelines by the World Medical Association Declaration of Helsinki 2000, 2004, 2008 and 2013 include additional specific areas of protection such as for the life, health, privacy, and dignity of participants [9, 24, 25, 38]. These terms used are still very broad, for example, protection of health or life. In 2002, the CIOMs provided guidelines which refer to the World Medical Association Declaration of Helsinki and Belmont report. Accordingly, the guidelines uphold that the researchers must make special provision for the protection of the rights and welfare of participants. However, the focus in the CIOMs is toward that of vulnerable individuals. While the 2002 and 2016 CIOMs guidelines focus on protection of vulnerable participants in research, the 2021 CIOMs guidelines include RCS as the main target for the protective duty of researchers.

In the context of RCS protection of research participants has become more complex and requires a more multifaceted and interconnected system of protection. In the protective duty, guidance documents ensures that participants welfare is of central concern to the researchers by minimising the level of harm to which participants may be exposed and treat them with respect and dignity throughout the study.

The exact structure of protective duty for research participants varies among guidance documents. Despite this flexibility, however, there are some basic protection functions necessary to ensure safety of participants, for example, protection against foreseeable study related harm, it is essential that researchers meet these needs. The empirical literature and evidence from research ethics guidance documents that exist on protection of study participants tend to show that this may only be meant for protection against study related harm. However, the researchers may extend this protection duty to incidental conditions identified during the study among their participants and provide the needed ancillary care.

Subjects may be reimbursed for lost earnings, travel costs and other expenses incurred in taking part in a study; they may also receive free medical services. It might, for example, be agreed to treat cases of an infectious disease contracted during a trial of a vaccine designed to provide immunity to that disease, or to provide treatment of incidental conditions unrelated to the study [7].

In addition to researchers’ duty not to harm participants in research, there is a duty to benefit participants where possible. Thus, where it is feasible for researchers to diagnose and treat an illness which arises, or to ensure that effective treatment is available at a local level, they have a duty to do so [28].

This call for the duty to provide ancillary care in essence can have protective benefits to study participants in RCS where they have several unmet health needs which may be more critical than the condition under study or as compared to the study related harm. Within the international ethics guidance documents from all years, explicit ancillary care obligation is not mentioned in the context of protecting study participants as one way of addressing their unmet health needs. Some participants may accept to participate in a study knowing that their needs will be taken care of and that they will be protected. It is suggestive of a strong belief that the moral grounds for such acts are dependent on the established relationship during the conduct of research. It is also suggestive of a strong belief that ancillary health care issues are a matter of personal responsibility, such that the researcher's obligation to protect the health of participants during research may be extended to include the provision of ancillary health care to their participants as a matter of personal responsibility.

There was limited discourse on protection of study participants from funding agencies and local research regulatory bodies guidance and policy documents beyond study related conditions, however, it was noted that the majority refer to the international guidance documents [4–7].

### Participant needs prevail over science

In this discourse, the key phrase is described in terms of researchers prioritising the responsiveness to the demands of participants in RCS. This discourse was first described in the 1989 World Medical Association Declaration of Helsinki [39] then later in CIOMS 1993, as cited in Nuffield Council on Bioethics [28], followed by the 1996 ICH-GCP [6] and is included in all the later versions of the World Medical Association Declaration of Helsinki, CIOMS, ICH-GCP. This textual phrase is also used in some policies from funding and policy organisations such as the MRC [40, 41], and the World Health Organization [42].

In research on man, the interest of science and society should never take precedence over considerations related to the wellbeing of the subject. [39]

Research in developing countries should be ‘responsive to the health needs and the priorities of the community in which it is to be carried out’ [CIOMS, 1993, as cited in [28]].

The rights, safety, and well-being of the trial subjects are the most important considerations and should prevail over interests of science and society [6].

In medical research on human subjects, considerations related to the well-being of the human subject should take precedence over the interests of science and society [24].

We observed that both international research ethical standards and funding agencies guidelines emphasise the importance of medical research not taking priority over participant demands. This discourse has been represented via the use of a variety of text phrases. The guidelines define the participants' interests as their well-being [6, 24, 42, 43], rights [6], safety [6, 43], health [42], and care [27, 42] (Table 1).

The goals of research, while important, should never be permitted to override the health, well-being, and care of research participants [42].

The safety and well-being of the individual prevail over the interests of science and society [43].

While some guidelines emphasise the importance of putting participants' interests above research and society, they do not specify whose participants' interests are being covered.

In all research involving people, an appropriate balance must be struck between the interests of participants (and, where relevant, the communities to which they belong) and the interests of society or the advancement of knowledge [44].

In a similar manner, this expression in the guidelines does not explicitly clarify whether it covers ancillary healthcare needs. While this may relate to the scientific information gained as a result of the study, it may also allude to the responsiveness of the research team to the participants' extra health requirements. Using more general phrases like well-being, health, and rights, does this suggest that researchers are responsible to provide care for the ancillary health needs of their participants?

The most common and important expectation of participants for ancillary care that researchers must meet, is to ensure the effacement of self-interest in placing the interests of their participants first. In biomedical research, however, commercialization of research participant protection has contributed significantly to the conflict between self-interest and ethical responsibility. This is particularly true in the situation of RCS in global south settings, where participants have a variety of extra health requirements that are left unmet by the health system. Consequently, although researchers have some ethical duties toward their participants during medical research, such as the need to protect their safety, they also have scientific interests that compete with the services that their participants are expecting at the same time.

### Supererogation

A third discourse on the researcher's role in the ethical conduct of research centred on behaviours that are morally praiseworthy but go above and beyond the call of duty in terms of research ethics. As defined by Jacobs [45], supererogation occurs when an agent performs activities that are morally right or morally praiseworthy, but which are not required by the actor's obligation. Even if particular acts fall short of what is objectively right, we should praise those who act from motives that are generally `utility maximising’ because praising such well-motivated acts tend to promote the best results [46]. The CIOMS’s earlier versions of 1993, as cited in Nuffield Council on Bioethics [28], 2002 [7] and 2009 [26] recognise ancillary care as an act that is commendable act to do for the participants but not required. Therefore, acts of ancillary care by researchers would be lacking in moral worth if they are not provided. This, on the other hand, does not serve as a guide for researchers, nor does it provide any legal framework under which researchers may be required to provide for the ancillary health care demands of their participants. The guidelines are explicit in stating that this is not a responsibility put on researchers, and that rather this is just an act of kindness. As such, the translation of this act into an obligation set out in guidance documents is not an imperative.

However, the CIOMS guidelines would appear to enshrine the research ethics guidance in the discussion of ancillary care responsibility:

Although sponsors are, in general, not obliged to provide health-care services beyond that which is necessary for the conduct of the research, it is morally praiseworthy to do so [7, 26].

Additional to this commitment, the guideline moves on to say; “in some circumstances, it may be relatively easy for researchers to treat the condition or refer participants to local health centre where treatment can be provided [7].” The phrase “morally praiseworthy” speaks to the researchers as the most powerful partners in the research-participant relationship. However, while the provision of any ancillary care is considered morally praiseworthy in the 2002 and 2009 CIOMS guidelines, no other international or national body has praised or recognized the provision of ancillary care or the researcher as being particularly "morally praiseworthy" for providing such services to their participants. According to a review of ethics guidance materials for both the local institutional review board [30, 31] and international funding agencies [40, 41, 44, 47, 48], there has been no evidence to suggest that such discourses of morally praiseworthy conduct are translated into institutional policies and guidance documents. However, we found that in almost all the guidance documents reviewed from the local regulatory and international funding institutions they refer to the Nuremberg code [2], the Declaration of Helsinki [24], the Belmont report [5], the ICH-GCP [6], and the CIOMS [24].

### Provide care as appropriate

While morally praiseworthy was used in the 2002 and other earlier versions of CIOMS guidelines, this phrase has been removed in the 2016 guidelines. Instead, the 2016 guidelines encourage provision of ancillary care as it may seem ‘appropriate or necessary’ by the researchers and other research stakeholders [8]. What is regarded suitable or required in this discourse seems to be dependent on the judgments that the researcher would make. So, similarly to the framing of ancillary care provision as ‘morally praiseworthy’ the statement "as it may seem appropriate or necessary" does not give any significant direction to researchers, particularly in RCS where every participant may have additional health-care requirements that qualify as being required or appropriate to provide care for. The Nuffield Council on Bioethics in their report which also serves as a guidance document on the ethical conduct of research particularly in developing countries and has been referenced by many other recent guidance documents including those for the international funding agencies such as the Wellcome Trust [44], also uses the phrase ‘if necessary’. However, the report encourages that researchers provide care for incidental finding among their participants if deemed feasible [28].

In addition to researchers’ duty not to harm participants in research, there is a duty to benefit participants where possible. Thus, where it is feasible for researchers to diagnose and treat an illness which arises, or to ensure that effective treatment is available at a local level, they have a duty to do so” [28].

The inclusion of the words `have a duty’ moves this provision from being an act of guidance, something that is ‘morally praiseworthy’ to being something that the researcher has an obligation to provide. However, like in the CIOMS 2016 guidelines, the Nuffield Council on Bioethics [28] report provides further guidance on what researchers can do when in that situation:

During research, participants may develop an entirely unrelated condition. In some circumstances, it may be relatively easy for researchers to treat the condition or refer participants to a local health centre where treatment can be provided. In other cases, researchers may not have the expertise to treat the condition effectively and appropriate treatment may not be available locally as part of the public health system [28].

The use of the passive term “may” in these guidance documents including some from funding agencies [49] suggests that funding agencies wish to give researchers options and not to make it obligatory, but this remains problematic in the sense that it does not provide an explicit position. The researcher in this case may be required to make decisions on a case-by-case basis as described in the 2016 CIOMS guidelines [8]. This has translated into funding guidance. For example, the Wellcome guidance notes on research involving people in low- and middle-income countries only emphasises that any considerations for the provision of ancillary care should be that which is equal to the local standard-of-care:

Where it is proposed to offer healthcare unrelated to the specific research question, we recommend that this should usually be the standard treatment that is available locally [44].

This is particularly problematic because there are disparities in standards of care between middle-income and low-income countries, as well as within those settings, and it contributes to further inequity, largely because there is a catch-all recommendation that is universalised without consideration for specific context.

### Ancillary care researchers’ obligation

The last and most recent discourse is about the ancillary care obligations researchers have towards their participants. The 2021 CIOMS guidance has been the first to clearly recognise ancillary care as an obligation of researchers toward their participants.

Researchers have an ethical obligation to care for participants’ health needs during research and, if necessary, for the transition of participants to care when the research is concluded [27].

These guidelines make some noticeable steps to demonstrate that researchers have a responsibility to care for their participants. This could be due to the fact that some researchers [50] have written on the conditions that the majority of RCS participants experience. These guidelines lay a strong focus on the fact that researchers have a commitment to provide ancillary care to their participants in order to assist them in addressing unmet health needs that remain unaddressed due to limited or unavailability of services in the local health care system, as stated in the guidelines. This discourse is suggestive of a social reality where the ethics of ancillary care during research places a greater value upon responding to participants needs. The guidelines provide further guidance that such care should not be considered as undue influence but rather that researchers should work to improve the health, quality, and access to health care services of their participants which are limited or not available.

While referral of participants requiring additional health care services from medical personnel with the necessary ability to continue the care (World Medical Association Declaration of Cordoba on patient-physician relationship), is supported in medical research guidance documents gives the same options to researchers. However, issues of limited availability of the required services are not well addressed. Just as with issues of standard-of-care, what if such services are limited or not available at all? This has not been well addressed in guidance documents particularly for the conduct of research in resource constrained settings.

### The translation of international guidelines to local research ethics guidance

While international research ethical guidelines include clear guidance for the provision care to study participants, we found limited guidance on the same from local research ethics guidelines and policy documents, which provide a significant research oversight. On the other hand, we found that the majority of local research ethics guidelines and policy documents are established pursuant to the International Ethical Guidelines for the Conduct of Research Involving Human Subjects, which are mostly regarded as primary source of guidance on research ethics matters.

These Guidelines have been developed basing on a number of resource materials including the Republic of Malawi Constitution; National Science and Technology Policy; National Procedures and Guidelines for the Conduct of Research in Malawi; Policy Measures for the Improvement of Health Research Co-ordination in Malawi; CIOMS; WHO Operational Guidelines for Ethics Committees That Review Biomedical Research; UNESCO Declaration on Bioethics and Human Rights, and other many relevant international ethical guidelines and regulations - Malawi National Health Sciences Research Committee [30]

The rights, safety and standards for research design and conduct are governed by the: Declaration of Helsinki, Nuremberg Code, and CIOMS [44]

These statements lay out a range of sources and options, demonstrating that the decision about which guidelines to follow is subjective. Lack of established local guidelines outlining the researchers' responsibilities towards their participants creates a gap when it comes to how researchers should respond to the additional health needs of their participants while participating in research studies. For example, the general guidelines on health research state that “medical care should be provided to research participants while they are participating in the study” Malawi REC Guidelines p. 22 [30], but no description is given of what that means or to what extent researchers can provide that care or when do such obligations stop. Such broad generalizations can confound researchers when designing their studies, which is particularly true in global south settings, where participants may have a variety of additional unmet health needs.

Despite significant progress in encouraging researchers doing studies in RCS to consider the provision of ancillary care by some funding agencies, there is limited attention on whether or not ancillary care should be considered to be an obligation by researchers. And, even if it did, there are no clear guidelines over how it should or could be monitored.

## Conclusion

Through this discourse analysis, the Nuremberg Code's ethics guidance, first published in 1947 and subsequently the declaration by the World Medical Association in 1964, demonstrates commitments and values towards the ethical conduct of research. The primary focus of these two first ethics guidelines, as well as all subsequent guidelines, is on protecting study participants against risk associated with research participation. The 1993 CIOMS guidelines and subsequent revisions in 2002, 2009, and 2016 established the concept of providing care to study participants during research, including for non-study-related diseases, known as ancillary care [11]. However, ancillary care was not acknowledged as a researcher obligation until the recent guidelines by the Council for International Organisations of Medical Sciences [27], which appears to reflect a sensitivity to the ethical need of researchers to provide ancillary care to their participants. This demonstrates a shift in the language away from a sole focus on the protection of study participants to one that includes the provision of additional care. While there have been major changes to the ethics guidance that reflect significant evolution in the ethical conduct of research, the specific vocabulary or language used to explain the ethics of researchers' ancillary care obligations to the health needs of their research participants is often complex and lacks clarity and consistency.

We acknowledge that this study has a limitation in that it is largely based on ethical guidance documents. We have not examined how such documents are implemented in practice, such as the actual procedure of ethics review by research ethics committees. Our analysis demonstrates how specific textual features guide researchers in both the global north and south to provide ancillary care to their study participants. Conducting additional qualitative methods research with research stakeholders in practice settings would provide insight into whether the shifts in language found within textual documents are reflected in current practices. That said, while this analysis is limited to ethics guidance documents, the research's broader message is applicable to guidance documents from funding agencies and local ethics bodies that do not provide explicit guidance on ancillary care.

Aspects of ancillary care are not currently standardised, as evidenced by several funding agencies' reluctances to express an opinion on the subject. Alternatively, it is possible that these funding agencies will defer to the researchers and the local research ethics guidelines in settings where the research is being conducted. However, these local research ethics guidelines also refer to international research ethics guidelines as described above, which leaves a gap on proper guidance on ancillary care provision. Additionally, this research found that, while discourses regarding the provision of care to study participants have evolved significantly over time, as demonstrated in the international ethics guidance documents, local ethics guidelines and policies of international funding agencies continue to refer to the Declaration of Helsinki of 2000, the Belmont report of 1978, the ICH-GCP of 1996, and the CIOMS of 2000. Due to a lack of explicit discourses on ancillary care in local research ethics guidelines and regulatory documents, research ethics committees have difficulty regulating or advising researchers regarding their ancillary care responsibilities. Additionally, we found that the current discourses used in international ethics guidelines, such as “morally praiseworthy,” “if necessary or as appropriate,” are too broad to serve as guidelines for researchers. Using such broad discourses fail to address general concern of ancillary care guidance on the extent to which this care can be provided and how does that apply to different contexts where medical research is conducted.

These historical depictions have a significant impact on the solutions that are proposed for health challenges faced by study participants in the global south, and as a result, we argue for explicit consideration of the ways in which writing choices on ancillary care can address some ethical issues in research. Our findings suggest that newer versions of ethics guidance documents must illustrate that the idea of ancillary care is explicitly included to provide researchers with clear guidance, particularly in RCS.

## Data Availability

Data sharing is not applicable to this article because all of the documents collected and analysed during the current study are already in the public domain. However, we are seeking to make available the documents we sourced via, CIOMS website (https://cioms.ch/), Wellcome Trust website (https://wellcome.org/grant-funding/guidance/research-involving-human-participants-policy), the H3Africa website (www.h3africa.org), NIH website (https://www.nih.gov/), MRC website (https://mrc.ukri.org/), ICH-GCP website (https://ichgcp.net/), World Medical Association website (https://www.wma.net/), and NCST website (https://www.ncst.mw/).

## Abbreviations

CIOMS: Council for International Organisations of Medical Sciences
COMREC: College of Medicine Research Ethics Committee
EDCTP: European & Developing Countries Clinical Trials Partnership
ICH-GCP: International Committee on Harmonization of Good Clinical Practice
MRC: Medical Research Council
NHSRC: National Health Sciences Research Committee
NIH: National Institute of Health
NIHR: National Institute for Health Research
RCS: Resource-constrained settings
REC: Research Ethics Committee
